# Advancing Migrant Access to Health Services in Europe (AMASE): Protocol for a Cross-sectional Study

**DOI:** 10.2196/resprot.5085

**Published:** 2016-05-16

**Authors:** Ibidun Fakoya, Débora Álvarez-del Arco, Susana Monge, Andrew J Copas, Anne-Francoise Gennotte, Alain Volny-Anne, Siri Göpel, Giota Touloumi, Maria Prins, Henrique Barros, Cornelia Staehelin, Julia del Amo, Fiona M Burns

**Affiliations:** ^1^ Centre for Sexual Health and HIV Research Research Department of Infection and Population Health University College London London United Kingdom; ^2^ National Centre of Epidemiology Instituto de Salud Carlos III Madrid Spain; ^3^ Biomedical Research Network on Epidemiology and Public Health (CIBERESP) Faculty of Political Science and Sociology Universidad Complutense de Madrid Madrid Spain; ^4^ Faculty of Medicine University of Alcalá Madrid Spain; ^5^ Department of Infectious diseases CHU Saint-Pierre Brussels Belgium; ^6^ European AIDS Treatment Group Brussels Belgium; ^7^ HIV Centre Frankfurt Frankfurt Germany; ^8^ Department of Hygiene, Epidemiology & Medical Statistics Medical School National and Kapodistrian University of Athens Athens Greece; ^9^ Department of Internal Medicine Division of Infectious Diseases Academic Medical Centre Amsterdam Netherlands; ^10^ Department of Infectious Diseases Public Health Service of Amsterdam Amsterdam Netherlands; ^11^ University of Porto Medical School Institute of Public Health University of Porto Porto Portugal; ^12^ Department of Infectious Diseases Bern University Hospital University of Bern Bern Switzerland; ^13^ Royal Free London NHS Foundation Trust London United Kingdom

**Keywords:** migrants, HIV, survey, community mobilization

## Abstract

**Background:**

Migrants form a substantial proportion of the population affected by the human immunodeficiency virus (HIV) epidemic in Europe, yet HIV prevention for this population is hindered by poor understanding of access to care and of postmigration transmission dynamics.

**Objective:**

We present the design and methods of the advancing Migrant Access to health Services in Europe (aMASE) study, the first European cross-cultural study focused on multiple migrant populations. It aims to identify the structural, cultural, and financial barriers to HIV prevention, diagnosis, and treatment and to determine the likely country of HIV acquisition in HIV-positive migrant populations.

**Methods:**

We delivered 2 cross-sectional electronic surveys across 10 countries (Belgium, France, Germany, Greece, Italy, the Netherlands, Portugal, Spain, Switzerland, and United Kingdom). A clinic survey aimed to recruit up to 2000 HIV-positive patients from 57 HIV clinics in 9 countries. A unique study number linked anonymized questionnaire data to clinical records data (viral loads, CD4 cell counts, viral clades, etc). This questionnaire was developed by expert panel consensus and cognitively tested, and a pilot study was carried out in 2 countries. A Web-based community survey (n=1000) reached those living with HIV but not currently accessing HIV clinics, as well as HIV-negative migrants. It was developed in close collaboration with a community advisory group (CAG) made up of representatives from community organizations in 9 of the participating countries. The CAG played a key role in data collection by promoting the survey to higher-risk migrant groups (sub-Saharan Africans, Latin Americans, men who have sex with men, and people who inject drugs). The questionnaires have considerable content overlap, allowing for comparison. Questions cover ethnicity, migration, immigration status, HIV testing and treatment, health-seeking behavior, sexual risk, and drug use. The electronic questionnaires, which were available in 15 languages, allowed for complex routing, preventing respondents from answering irrelevant questions.

**Results:**

In total, we recruited 2249 participants from 57 HIV clinics as part of the clinic survey and retrieved 1637 complete responses as part of the community survey.

**Conclusions:**

The findings will provide much-needed information for improving HIV prevention interventions and access to services for migrant communities.

## Introduction

In 2014, with new human immunodeficiency virus (HIV) infections on the decline, the Joint United Nations Programme on HIV/AIDS (UNAIDS) announced the beginning of the end of the AIDS epidemic [1]. Although there are still approximately 35 million people living with HIV worldwide, UNAIDS has set a goal of ending the AIDS epidemic in “every region, every country, in every location, in every population and every community” by 2030 [1]. Reaching this ambitious goal is now possible because of vast improvements in the ability to provide widespread HIV testing and subsequent treatment with antiretroviral therapy. Within Europe, the end of AIDS is dependent on identifying, treating, and preventing onward transmission among an estimated 2.2 million people living with HIV [2], a substantial and disproportionate number of whom are people who were born in another country, that is, migrants. While 9.7% of the European Union (EU)/European Economic Area population were born outside the current borders of their country of residence [3], over a third (35.0 %) of those newly diagnosed with HIV in 2013 were migrants [4]. Approximately 15% of those were people who had migrated from a country with a generalized epidemic, notably sub-Saharan Africa (13%). Smaller proportions of newly diagnosed migrants were from Central and Eastern Europe (5.1%), Latin America and the Caribbean (4.9%), other Western European countries (3.9%), and South and Southeast Asia (2.2%) [4].

Despite the heavy burden of HIV among migrant communities, HIV prevention and treatment for these populations is hindered by a relatively sparse evidence base and the heterogeneity of the populations affected [5]. Migrants are more likely to be exposed to social determinants of ill health (such as poverty, social exclusion, and unemployment), which can result in poor health-seeking behaviors [6]. Other structural factors such as legal and administrative status, together with health care- and community-related barriers, can further prevent migrants from accessing health services, particularly HIV testing [7]. Yet little is known about migrant access to health services, particularly HIV testing, access to treatment, and safer sex practices [8]. Previously it had been assumed that most HIV diagnosed among migrants in Europe was acquired in the country of origin, particularly those born in sub-Saharan Africa. Yet it is unclear whether infections are acquired pre- or postmigration [5]. Although surveillance data show that people living outside their country of birth form a substantial proportion of the population affected by the HIV epidemic in Europe, limited data are available about migrant populations to inform policy and practice for these communities.

Understanding barriers to accessing HIV care and postmigration transmission dynamics will provide policy makers and program managers much-needed evidence for effectively planning HIV prevention programs for migrant communities. Several cross-sectional sexual health and HIV studies have sampled migrant populations, but these were often limited to migrants from 1 region (black Africans in Burns et al [9] or Central and Eastern Europeans in Evans et al [10]) or migrants in 1 country (Dray-Spira et al [11]), and there has not been a collaborative European study to address these research questions jointly. Within the European Network of HIV/AIDS Cohort Studies to Coordinate at European and International Level Clinical Research on HIV/AIDS (EuroCoord), we set up the advancing Migrant Access to health Services in Europe (aMASE) study whose overall aim is to provide the evidence to prevent HIV infection and improve diagnosis and prognosis of migrant populations living with HIV in Europe in order to support policy development at the European level. Specifically, aMASE aims to identify the structural, cultural, and financial barriers to HIV prevention, diagnosis, and treatment and to determine the likely country of HIV acquisition in HIV-positive migrant populations. In this paper, we present the design and methods of the 2 cross-sectional studies that form aMASE and the unique challenges associated with multisite, multidisciplinary, multinational research.

## Methods

### Study Design

aMASE is formed of 2 cross-sectional, electronic surveys of 2 populations: (1) migrant adults living with HIV and attending HIV services (the clinic survey), and (2) migrant adults living in Europe (the community survey). For the purposes of this study, we define migrants as foreign-born individuals intending to live in their current country of residence for ≥6 months. Residency was not dependent on formal documentation, and immigration status, while captured within the survey, was not part of the inclusion or exclusion criteria. The clinic survey was delivered using a computer-assisted self-interview (CASI) or computer-assisted personal interview (CAPI) and augmented with clinical data from patient records. The community study was a Web-based survey designed to reach those living with HIV but not currently accessing HIV clinics, as well as HIV-negative migrants.

### Clinic Survey

#### Study Setting and Time Period

The clinic survey was implemented in 57 clinics in 9 countries (Belgium, Germany, Greece, Italy, the Netherlands, Portugal, Spain, Switzerland, and the United Kingdom) between July 2013 and June 2015. Multimedia Appendix 1 lists the clinics’ names.

#### Participants and Eligibility Criteria

Patients were eligible for inclusion in the study if they were (1) HIV-positive, (2) aged ≥18 years, (3) foreign-born and resident in the country of recruitment for ≥6 months, (4) diagnosed within 5 years of the study date, and (5) able to complete, either alone or supported, a CASI in any of the 15 languages available (Amharic, Arabic, Dutch, English, French, German, Greek, Italian, Polish, Portuguese, Russian, Turkish, Tigrinya, Spanish, and Somali). In Switzerland, local eligibility criteria applied (migrants from neighboring Austria, France, Germany, and Italy are excluded due to close linguistic and cultural ties).

#### Sample Size

The sample size calculation was based on precision and used the standard formula for standard error of a proportion: SE=Sqrt (p*(1–p)/n). In calculating the sample size, the primary outcome measures were health service use (registered with primary care physician) and the proportion of participants with a previous negative HIV test.

Initially we anticipated recruiting 4000 participants (2000 men and 2000 women) from 2 to 5 clinics in each of 7-9 EU countries (creating about 40 clusters). We considered the within-cluster correlation likely to be relatively weak (eg, 0.005), at least for regression analyses, as the average cluster size may potentially have been large (eg, 75-125). Assuming a design effect of 1.5 for the study, the overall effective sample size would have been ~2670. This sample size would have provided good precision for our estimates. Specifically, within each gender group, our outcomes could be estimated to within 3% across Europe and to within around 8% for each country. As recruitment was slower than anticipated, to a large extent due to the decrease in the number of new HIV-positive migrants in Europe [12], we revised this sample size in December 2014.

The revised target sample size calculation was 2000 participants (1000 men and 1000 women) from all clinics. We recruited participants from a minimum of 2 clinics in each of the 9 countries, with each clinic forming a discrete cluster. We estimated this within-cluster correlation also to be relatively weak (eg, 0.005), at least for regression analyses including patient and city characteristics as covariates. As the average cluster size was likely to be smaller than first estimates (eg, 40–60), we assumed a design effect of 1.25 for the study and, hence, an overall effective sample size of ~1600. This revised sample still provides good precision for estimates. Specifically, within each gender group, we will be able to estimate our outcomes to within 3.5% across Europe and to within 10% for each country.

#### Sampling Strategy

The main inclusion criteria for study sites were a sizable migrant clinic population (sufficient to recruit a minimum of 40 study participants in most sites) and the human resource capacity to conduct the study with minimal additional funding. Eligible patients attending participating clinics within the study period were approached by members of their clinical care team or a recruitment researcher to participate in the clinic survey.

#### Variables and Questionnaire Development

We formed a working group, made up of international experts and EuroCoord collaborators, to act as an expert panel tasked with reaching consensus on survey instrument development and provide overall supervision of the study. Where possible, we adapted questionnaire items from existing survey instruments. New questions were drafted by the core research team and expert panel members.

We validated the questionnaire using cognitive testing (cognitive aspects of survey methodology approach [13]). It was tested in Spanish (in Spain) and English (in the United Kingdom) on 7 black African and 3 Latin American migrants recruited from community-based HIV service organizations. The finalized patient questionnaire is divided into 3 sections: (1) detailed sociodemographic data and extensive migration history data, (2) sexual and HIV risk behavior, including drug use before and after migration, and (3) service use and experiences of living with HIV, including stigma and discrimination. Questions were tailored to reflect the different health care or ethical approval systems in different countries. For example, respondents completing the survey in Portugal were not asked questions about ethnicity or health care costs. There are 90-92 items included depending on current country of residence, although through skipping and filters some respondents answered as few as 43 questions. Most questions were closed and the survey completion time was between 15 and 50 minutes depending on the language selected. The survey was translated from English into 15 languages (see Participants and Eligibility Criteria above) by a professional translation company. These translations were then checked by a different translation company, who back-translated a portion of the questionnaire into English to ensure quality control.

Participating patients’ questionnaires were matched to a clinical data form using a unique study number. The clinical data form contains 20 items, including CD4 cell count and viral load (at diagnosis, at antiretroviral therapy initiation, and the latest available); previous HIV-negative tests and viral clade; AIDS-defining illnesses and coinfections; and treatment initiation. Clinical data were completed electronically.

#### Ethics

Ethical approval for the aMASE studies was received separately in each participating country (see Table 1).

#### Pilot Study

We piloted the function and reliability of the CASI and the clinical data form in 3 clinics (2 in London, 1 in Madrid) with 115 patients. The pilot study also tested whether recruitment procedures, study implementation, and data collection, storage, and handling methods were feasible and appropriate. Following feedback about the function of the CASI software from recruitment researchers, we redesigned the survey using new CASI software. To facilitate recruitment, the selection criterion of HIV diagnosis in the previous 3 years was increased to 5 years. Based on the results of the pilot study, the survey questions remained essentially the same; however, we redesigned some items to produce a better user experience.

#### Study Implementation

The patient questionnaire was administered using tablets, computers, or laptops running Fluidware version 5.0 (SurveyMonkey Canada Inc, Ottawa, ON). Enrollment commenced in participating clinics in July 2013 and was completed in July 2015.

As of August 2015, there were 57 participating clinics in 9 countries: Belgium (n=4), Germany (n=2), Greece (n=8), Italy (n=2), the Netherlands (n=3), Portugal (n=7), Spain (n=18), Switzerland (n=6), and the United Kingdom (n=7).

A study coordinator at the Institute of Health, Carlos III, Spain, was responsible for the overall management of the fieldwork, but in each country a nominated country lead (usually a member of the expert panel) was responsible for collating data across clinics in his or her country. This coordinator actively engaged with all participating centers and country leads to ensure compliance with the study standards, and identified aspects that needed improvement throughout the study period. Where necessary, clinics were supplied with laptops and tablets preloaded with CASI software and instructions for use. Clinics were given a clinic study pack (developed during the pilot study), which detailed the general protocol and maintenance instructions for the CASI devices. Clinic recruiters were able to access an online Web resource with electronic versions of all the required documentation for the study. Incentives were not provided to participants, although some clinics supported travelling costs in the context of their research practices.

Recruitment procedures varied slightly across countries and between clinics, but in general recruitment and consent procedures were as follows.

Eligible patients were identified though clinic databases and their clinical records were flagged. Clinic numbers of eligible patients were noted next to precoded unique study numbers on an enrollment log. Eligible patients were then approached by either members of their clinical care team or a recruitment researcher and provided with a patient information sheet and the opportunity to ask questions. Patients who declined to participate had their age, ethnicity or nationality, and gender noted on the enrollment log. Those who agreed to participate were asked to complete the CASI/CAPI on site. In most countries, ethical approval for obtaining informed consent through a tick box in the questionnaire was given; however, in Belgium, Switzerland, Greece, and Germany local ethical approval standards required separate consent forms.

Enrollment logs were stored in a locked cabinet; electronic versions of the completed forms were returned to the study coordinator on a monthly basis. Survey data were either captured and then stored directly on a central secure database operated by FluidSurveys (online questionnaire) or stored on the device’s hard drive until they were transferred to a secure network in Madrid (offline questionnaire).

####  Statistical Analysis

We will explore barriers to accessing health care by using the primary outcomes measures: access to a primary care physician and a previous negative HIV test.

Participating clinics are not necessarily representative of the number of clinics within countries or, indeed, the migrants living with HIV within those countries. For these reasons, the overall sample prevalence of our primary outcome measures, pooled across countries and clinics, may not be informative. We shall give it emphasis in our results only if the prevalence across countries is similar and otherwise focus on the prevalence in each country. We anticipate that associations with the primary outcomes, for example their association with age, will be broadly comparable across countries. However, if associations differ substantially between countries, then we shall investigate this heterogeneity and report an overall measure of association if we judge this to be meaningful. Furthermore, as men and women are expected to have different barriers to accessing care, we will analyze them separately. We will analyze individuals who identify as transsexual separately if numbers are sufficient. We will conduct analyses separately by region of birth if associations differ substantially by region, and we may conduct separate analyses for men who do and do not have sex with men if associations differ substantially between these 2 groups of participants.

Our interest is in associations between the primary outcome measures and each of the following factors: age, gender, region of birth, sexual orientation, immigration status, and age at migration or diagnosis. We will first describe these associations through cross-tabulations. To acknowledge the clustering of participants, we shall analyze associations using hierarchical random effects logistic regression, with a random effect for clinics nested within countries, analyzing factors individually and then jointly in a multiple regression model. We will report associations as odds ratios with 95% CIs.

Within EuroCoord, a group of experts has been established to develop and evaluate a reliable algorithm to determine probable country of infection. The algorithm will incorporate demographic (eg, gender, race or ethnicity, age) and clinical data (eg, CD4 counts, HIV RNA levels, clinical stage according to the US Centers for Disease Control and Prevention) and will be evaluated in simulation studies and in cohorts with well-estimated seroconversion dates. The algorithm will be further elaborated using data from the questionnaire (eg, migration and HIV testing history, sexual partners pre- and postmigration). CD4 cell counts and viral loads for the study participants will be extracted from the clinical records. Methods for calculating a proxy date for seroconversion rely on clinical parameters with very little, if any, behavioral information informing the model [14]. Our analysis will draw on more detailed information than what is available in routinely collected surveillance data, thus allowing for a more nuanced approach in determining probable country of infection. We will use imputation methods to account for missing data in clinical records. Further details on the methodology used to develop, verify, and apply the algorithm will be published separately.

#### Response Rates

The age, nationality or ethnicity, and gender of all patients approached to participate in the study were noted on a clinic log. We will analyze these data to assess whether those who agreed to participate are significantly different from those who declined.

### Community Survey

#### Study Setting and Time Period

We designed the aMASE community survey to complement the clinic survey and capture data on all migrants regardless of their HIV status. The community survey was available through Web browsers between April 2014 and July 2015.

#### Participants and Eligibility Criteria

All migrants living in the World Health Organization European area (52 countries) aged ≥18 years, irrespective of their HIV status or current country of residence, were eligible to participate in the aMASE community survey.

#### Sample Size

No sampling frame for migrants in Europe exists, so we used a convenience sampling strategy. The main outcome measures on which we based our sample size estimates were registration with a primary care physician and ever having tested for HIV. The community survey aimed to recruit 1000 migrants, which allows for estimates of outcomes by gender to within 5%. This sample size also provided 80% power to detect a difference of 6% in 1 of the key outcomes (assuming an overall outcome prevalence of 50%) compared with service users (in the clinic study), or smaller differences for less prevalent outcomes.

#### Sampling Strategy

We actively promoted the survey from June 2014 to May 2015 using social marketing and community participatory methods in 9 countries: Belgium, France, Germany, Greece, Italy, the Netherlands, Portugal, Spain, and the United Kingdom. We targeted some of these countries for active promotion because they were also involved in clinic recruitment. An ongoing survey with African migrants in Switzerland precluded active promotion in that country, and we selected France because it has a large migrant population. A community advisory group (CAG), a group of individuals working for local community-based organizations that provide services to migrant communities or oversee pan-European migrant or HIV networks, was integral to the sampling strategy. The aMASE expert panel selected CAG members with representation from all countries involved in active promotion of the survey. CAG members were contracted to deliver outreach meetings, with the aim of promoting the survey to other organizations within their country. In turn, these organizations promoted the survey to other organizations and their service users and cascaded the study promotion in a method similar to snowball sampling.

#### Variables and Questionnaire Development

We developed the community survey instrument using an iterative community participatory approach involving the CAG and the expert panel. The expert panel was responsible for the survey content and design and all technical aspects of administering the questionnaire. The CAG ensured that the survey content was relevant to migrant communities within Europe and provided a “real-world” critique of the survey items. The CAG was able to highlight culturally insensitive questions (eg, increasing the number of available gender categories from 2 to 6) or request items that will provide findings that can be quickly translated into policy and practice recommendations (eg, knowledge about access to free condoms). After 4 iterations of the survey development process, we tested the survey CASI for programming inconsistences. The survey was then translated into 14 languages (identical to the clinic survey with the exclusion of Tigrinya) by a professional translation company. These translations were then checked by members of the CAG or colleagues fluent in the available languages. Almost three-quarters (66/93) of the items in the clinic survey patient questionnaire are replicated in the community questionnaire to allow for comparison between the 2 samples. The remaining items include country-specific questions and topics of interest to the CAG and cover health service use in the country of origin or residence; home testing or sampling for HIV and other infections; mental health; and social determinants of health (eg, housing).

**Table 1:** Ethical approval for the advancing Migrant Access to health Services in Europe (aMASE) study in each country.

Country	Committee	Number
Belgium (Antwerp)	Institute of Tropical Medicine, Institutional Review Board	911/13
Belgium (Brussels)	Comité local d’éthique hospitalier, Centre Hospitalier Universitaire Saint-Pierre	B076201215754
Belgium (Gent)	Universitair Ziekenhuis Gent, Commissie voor Medische Ethiek	B076201215754
Belgium (Liège)	Comité local d’éthique Hospitalo-Facultaire Universitaire de Liège	B707201318791
Germany (Bonn)	Rheinische Friedrich-Wilhelms-Universität Bonn, Medizinische Fakultat Ethik-Kommission	008/14
Greece	National and Kapodistrian University of Athens Institutional Review Board	6/3/2013^a^(# 6313)
Italy	Istituto Nazionale per le Malattie Infettive “Lazzoro Spallanzani”	22/02/2013^a^
The Netherlands	Universiteit van Amsterdam	2013_137#C20131038
Portugal	Centro Hospitalar de São João, EPE	28/8/2013^a^
Portugal	Hospital Prof. Doutor Fernando Fonseca, EPE	31/8/2013^a^
Portugal	Centro Hospitalar Lisboa Norte, EPE	9/10/2013^a^
Portugal	Centro Hospitalar Lisboa Central, EPE	11/7/2013^a^
Portugal	Centro Hospitalar de Setúbal, EPE	21/1/2015^a^
Portugal	Comissão Nacional de Proteção de Dados (Portuguese Data Protection Authority), EPE	14/10/2014^a^
Spain	Comité de Ética de la Investigación y del bienestar animal, Instituto de Salud Carlos III	CEI PI 01_2012-v2
Switzerland	Kantonale Ethikkommission Bern	024/13
United Kingdom	London-Bentham Research Ethics Committee	11/LO/1600

^a^Date of approval letter.

#### Ethics

Ethical approval was obtained from the London Bentham Research Ethics Committee (11/LO/1600). Additional approvals were obtained in all countries also undertaking the clinic survey.

#### Pilot Study

We carried out a short pilot study with CAG and expert panel members (38 respondents) who completed the survey in a language of their choice. The CASI was revised to improve the quality of translations and make them more suitable for our target population.

#### Study Implementation

EuroCoord’s primary community partner, the European AIDS Treatment Group (a European network of nationally based volunteer activists), employed a project coordinator to assist the research team in community engagement and chair the CAG. CAG members were provided with a suite of promotional materials including posters, business cards, postcards, and electronic banner advertisements, which were available from the aMASE website. Over 18,000 small media items (3100 business cards; 15,600 postcards) were printed and disseminated to community-based organizations across Europe. The CAG also used additional promotional and recruiting strategies: for example, 12 organizations (in France, the Netherlands, Greece, and the United Kingdom) allowed participants to complete the survey on laptops or other devices on their premises.

We contacted a further 243 community-based organizations identified through a mapping exercise and asked them to promote the survey on their websites and social media pages (eg, Facebook, Twitter). Approximately 300 webpages and apps also carried banner advertisements or links for the community survey, including PlanetRomeo, Grindr, Poz Traveler, and EasyExpat.

All community survey promotional materials referenced the aMASE website, where potential participants were able to select 1 of the 14 survey languages on the initial landing page [15]. Participant information sheets about the community survey were available in all 14 survey languages. Further information with in-depth details about both cross-sectional surveys was available in Dutch, English, French, German, Italian, Portuguese, and Spanish. Individuals who wished to participate in the study clicked through to a separate website, where the survey is hosted by FluidSurveys. All participants provided within-survey tick box consent. Survey data were captured and then stored directly on a central secure database operated by FluidSurveys.

####  Statistical Analysis

Statistical analysis will follow similar methods to those outlined for the clinic survey. Direct comparisons between the clinic population and the community population in the primary outcome measures may identify additional barriers to accessing health care, especially HIV testing, treatment, and care. Secondary outcomes explored in the community survey include chronic illness and infection other than HIV; access to condoms; access to needle exchange programs; and experiences of racism and homophobia.

### Funding

The aMASE study is part of Work Package 14 of EuroCoord, which is funded by the EU’s Seventh Framework Programme for research, technological development, and demonstration (under grant no. 260694). The community mobilization and engagement project was sponsored by Gilead Sciences Europe Ltd. Multimedia Appendix 2 lists other funding sources that contributed to the aMASE study.

## Results

By the end of July 2015, recruitment for both clinic and community surveys had concluded. In total, we recruited 2249 participants from 57 HIV clinics as part of the clinic survey and we retrieved 1637 complete responses as part of the community survey. Following data cleaning and analysis, we submitted initial findings and results for peer review in April 2015.

## Discussion

The aMASE study is the first multinational study to specifically sample migrants from across the globe living in Europe. The data gathered will provide not only valuable information about the barriers faced by migrants when trying to access HIV testing, prevention, treatment, and care, but also data about postmigration HIV acquisition. A literature review estimated that postmigration HIV acquisition among migrants from countries with a generalized epidemic range from 2% in Switzerland to 62% among migrant black Caribbean men who have sex with men in Europe [5]. The aMASE study will be the first to estimate postmigration HIV acquisition among European migrants living in Europe, as well as those from endemic regions. These data will provide policy makers and HIV program managers with a sound evidence base on which to develop HIV testing and prevention initiatives for these populations.

There were limitations to this study. Clinics from 9 countries were involved in data collection for the clinic study, and we recruited participants from 57 clinics. We selected these countries in part because they have large migrant populations (including those from Latin America and sub-Saharan Africa); in 2011, the United Kingdom, Germany, Spain, and Italy were the destination countries of 60.3% of all immigrants to EU member states [3]. The selected countries also contain the largest numbers of nonnationals (not including migrants who acquired citizenship in their current country of residence) living in the EU: Germany (7.7 million persons), Spain (5.1 million), Italy (4.4 million), the United Kingdom (4.9 million), and France (3.8 million) [3]. In Greece and Belgium, migrants make up a substantial proportion of the population (7.8% and 11.2%, respectively). Migrants living in France are a notable omission from our clinic sample; however, agreements are in place that will enable us to compare aMASE data with data from similar studies conducted in France.

Participating clinics were not chosen at random and may not be representative of clinics throughout their country of location. Nonetheless, the majority of sites are located in conurbations with large migrant populations, and the clinic sample is likely to be broadly representative of migrants accessing health care within each participating country. Our clinic (and community) sample is unlikely to be representative of the European migrant population as a whole in terms of such factors as country of residence, region of birth, gender, and sexual orientation. Consequently, a naive analysis of the overall data might underestimate or overestimate the strength of associations with our outcomes in the total population, but we believe our strategy of investigating heterogeneity of associations between countries and conducting analyses separately within subgroups determined by these factors, where necessary, will limit the bias in our findings.

Finally, the long recruitment period for both the clinic and the community surveys may present a challenge to interpreting the data. Migrant populations within Europe can fluctuate and change rapidly. Those recruited at the beginning of the study may represent a different migration cohort with differing barriers to accessing care from those recruited toward the end.

The aMASE clinic survey links self-reported data provided by participants with data from clinical records, which is vital for the accurate estimation of probable country of HIV acquisition. Furthermore, the clinical data will also allow us to estimate factors associated with late diagnosis, recent seroconversion, and hepatitis coinfection, thereby providing insight into the barriers and facilitators to earlier testing and access to care for study populations that have rarely been the sole focus of HIV research in Europe. The clinic survey will also augment the evidence base with regard to distal determinants of health such as poverty and hunger, as well as the sexual health needs of migrants living with HIV.

The aMASE Study as a whole also provides a useful model for community participatory methods in research. The community survey was developed using iterative methods with close communication between the research team, a panel of experienced epidemiologists, statisticians, and HIV clinicians, and an advisory group made up of community activists. Actively involving the CAG in survey development was time consuming and presented logistical challenges for the research team (eg, language barriers, inability to schedule some meetings during the working day). Those issues notwithstanding, the benefits in adopting this strategy were clear shortly after the 2014 European parliamentary elections, in which parties that campaigned on an anti-immigration platform performed successfully. Without the endorsement and support of the CAG, a study about the health of migrants in Europe may have been viewed with suspicion across the political spectrum. A careful balancing act between active promotion on migrant-friendly media and low-level promotion on mainstream media has enabled the CAG and the research team to promote the study without experiencing a significant backlash from anti-immigrant organizations.

Community stakeholders will be actively involved in guiding analysis and disseminating the results of both surveys. A detailed publication agreement has been drafted that gives community-based organizations access to community survey data relevant to their communities. In addition, CAG members will assist in creating a policy advocacy information sheet based on the results of both surveys. This information sheet will provide community-based organizations with key local and pan-European health policy advocacy points designed to improve access to health care for people living with diagnosed HIV. Involving community stakeholders in this final stage of the study increases the likelihood that the findings from this study will be incorporated into policy and practice across Europe. The results of this study will improve the understanding of postmigration transmission dynamics and the barriers to health care for migrants in Europe.

## Appendix

Multimedia Appendix 1 aMASE Clinic Survey Sites.

Multimedia Appendix 2 aMASE Funding Sources.

## Abbreviations

aMASE: advancing Migrant Access to health Services in Europe
CAG: community advisory group
CAPI: computer-assisted personal interview
CASI: computer-assisted self-interview
EU: European Union
EuroCoord: European Network of HIV/AIDS Cohort Studies to Coordinate at European and International Level Clinical Research on HIV/AIDS
HIV: human immunodeficiency virus
UNAIDS: Joint United Nations Programme on HIV/AIDS
