# Supplementary material for: Advancing Migrant Access to Health Services in Europe (AMASE): Protocol for a Cross-sectional Study
Source: JMIR Res Protoc. 2016 May 16;5(2):e74. doi: 10.2196/resprot.5085 (PMC4886100; doi:10.2196/resprot.5085)
Supplement: Multimedia Appendix 1 [file resprot_v5i2e74_app1.pdf]

**Belgium**	
Country Leads & Coordinators	Anne-Françoise Gennotte, Cécile La Morté
Ghent University Hospital	Aerssens A (they has to send us the PI name)
HIV/AIDS Center, Institute of Tropical Medicine, Antwerp	Nöstlinger C, Manirankunda L, Van Frankenhuijsen M
CHU Sart Tilman	Moutschen M, Dellot P, Hermans I
Department of Infectious Diseases, St Pierre University Hospital, Brussels	Clumeck N, Gennotte AF, La Morté C, Barthélemy A
**Germany**	
Country Leads & Coordinators	Gundolf Schüttfort, Claudia Wengenroth
Universitätsklinikum Bonn	Rockstroh JK, Wasmuth JC, Schwarze- Zander C, Boesecke C, Mohrmann K, Engelhardt A
Universitätsklinikum Frankfurt	Goepel S, Schuettfort G, Stephan C, Ebeling F
**Greece**	
Country Leads & Coordinators	Giota Touloumi, Gountas Ilias, Koulai Loumpiana, Anagnostou Olga
Infectious Diseases & HIV Division, Dept. of Internal Medicine, Evaggelismos Athens General Hospital	Skoutelis A, Papastamopoulos V
AIDS Unit, Clinic of Venereologic & Dermatologic Diseases, Athens University, Medical School, Syngros Hospital	Paparizos V, Kourkounti S
1st Dept. of Propedeutic Medicine, Athens University, Medical School "Laikon" General Hospital	Daikos G, Psichogiou M
"Attikon" University General Hospital	Antoniadou A, Protopapas K
1st Dept. of Medicine, Infectious Diseases Unit, "G. Gennimatas" Athens General Hospital	Gargalianos-Kakolyris P, Loyrida G
1st Dept. of Internal Medicine, Infectious Diseases Devision, AHEPA University Hospital, Aristotle University HIV Unit	Metallidis S, Tsachouridou O
1st Dept of Internal Medicine, Infectious Diseases Section, Patras University Hospital	Gogos HA, Leonidou L
**Portugal**	
Country Leads & Coordinators	Henrique Barros, Paula Meireles
Hospital de Curry Cabral	Maltez F, Fortes AL, Lino S, Cardoso O, Cabo J, Pinheiro A
Hospital de Santa Maria	Valadas E, Sutre AF, Boura M
Hospital de Santo António dos Capuchos	Almeida A, Mendes A
Hospital de São Bernardo	Poças J, Silva V, Rodrigues E, Feijó M
Hospital de São João	Serrão R, Piñeiro C, Soares J, Caldas C
Hospital de São José	Germano I, Martins L, Moura TS, Azevedo L, Fevereiro C, Mata-Mouros P
Hospital Prof. Doutor Fernando da Fonseca	Pacheco P, Cunha J, Lopes MJ
**Spain**	

Country Leads & Coordinators	Julia del Amo, Débora Álvarez, Cayla J, Garcia de Olalla P, Ospina JE
Centro Sanitario Sandoval	del Romero J, Rodríguez M, Vera M, Río I, Paredes V, Sanz N
Hospital Ramón y Cajal	Dronda F
Hospital de Alcorcón	Velasco Arribas M
Hospital de Sant Pau	Mateu MG, Gutierrez MM, Domingo P
Hospital del Mar	Knobel H, Pellicer T
Hospital Clínic	Fernández E, Ligero MC, Robau M, Miró JM
Hospital de la Vall d'Hebron	Ocaña I, Burgos J
Unidad de ITS de Drassanes	Barberà MJ, Arando M
Hospital de Elche	Gutiérrez F, Masía M
Hospital San Pedro de La Rioja	Blanco JR
CIPS Alicante	Belda-Ibáñez J, Fernández García E, Zafra Espinosa T
Hospital de Poniente	Lopez Lirola A
Hospital La Fe	Salavert M, Montero M, Calabuig E, Cuellar S
Hospital Virgen del Rocío	Vinciana P, Palacios R
Hospital San Cecilio	García F, Peña A
Hospital Donosti	Iribarren JA, Aguado M
Hospital Doce de Octubre	Pulido F, Portillo A
Hospital Universitario Canarias	Gómez Sirvent JL
**Switzerland**	
Country Leads & Coordinators	Bruno Ledergerber, Cornelia Staehelin
Bern University Hospital	Staehelin C
University Hospital Geneva	LeCompte T
University Hospital Zürich	Ledergerber B, Fehr J
University Hospital Basel	Thierfelder C
University Hospital Lausanne	Darling K
Cantonal Hospital St. Gallen	Bertisch B
**The Netherlands**	
Country Leads & Coordinators	Maria Prins, Janneke Bill, Freke Zuure
Academic Medical Center	Prins JM
Onze Lieve Vrouwe Gasthuis	Hoeksema K
Medisch Centrum Haaglanden	Leyten EMS
Stichting HIV Monitoring	Reiss P
**Italy**	
Country Leads & Coordinators	Tullio Prestileo , Nicolla Petrosillo
Spallanzani Medical Center	Petrosillo N

Ospedale Civico-Benfratelli Palermo	Prestileo T, Corrao S, Prestileo F
**United Kingdom**	
Country Leads & Coordinators	Fiona Burns, Ibi Fakoya
Birmingham Heartlands Hospital	Taylor S, Gilleran G, Stretton C
Guys and St Thomas’	Fox J, Patel N, Melling P
Homerton University Hospital	Anderson J, Mguni S, Sanjani C
King’s College Hospital	Post F, Campbell J, Engler B, Yurdakul S, Okumu-Fransche S
North Manchester General Hospital	Wilkins E, Lindegard G
Mortimer Market Centre	Gilson R, Milinkovic A, Tudor K, Young C
Royal Free Hospital	Johnson M, Fernandez T,
