# Supplementary material for: Advancing Migrant Access to Health Services in Europe (AMASE): Protocol for a Cross-sectional Study
Source: JMIR Res Protoc. 2016 May 16;5(2):e74. doi: 10.2196/resprot.5085 (PMC4886100; doi:10.2196/resprot.5085)
Supplement: Multimedia Appendix 2 [file resprot_v5i2e74_app2.pdf]

- European Union’s Seventh Framework Programme for research, technological development and demonstration under EuroCoord grant agreement n° 260694
- National Institute of Health Research Doctoral Research Fellowships, United Kingdom (Ibidun Fakoya)
- Gilead Sciences Europe (Community Mobilisation)
- National Institute of Health Research Clinical Research Network, United Kingdom (UK clinical recruitment)
- Foundation for AIDS research and prevention in Spain (FISPSE) Proyect 361036/10: (Likely Country of Acquisition of HIV infection and Barriers to access to prevention and HIV testing of migrant population in Spain)
- Consortium of Biomedical Research in Epidemiology and Public Health, Spain (Ciberesp).
- Spanish HIV Research Network for Excellence (RD06/006) and RD12/0017/0018
- Research and Development Fund, Public Health Service of Amsterdam, The Netherlands (recruitment in the Netherlands)
- aMASE in Switzerland was jointly funded by EuroCoord and the Swiss HIV Cohort Study (www.shcs.ch)
